# Oral ATP administration improves blood flow response to exercise in an animal model and in resistance trained athletes

**DOI:** 10.1186/1550-2783-10-S1-P16

**Published:** 2013-12-06

**Authors:** Ryan P Lowery, Michael D Roberts, Frank W Booth, Jordan Joy, Clayton L Cruthirds, John A Rathmacher, Shawn M Baier, John C Fuller, Christopher M Lockwood, Ralf Jäger, Joshua E Dudeck, Jacob M Wilson

**Affiliations:** 1 Department of Health Sciences and Human Performance, The University of Tampa, Tampa, FL, USA; 2 Department of Biomedical Sciences, College of Veterinary Medicine, University of Missouri, Columbia, MO, USA; 3 Department of Nutrition and Exercise Physiology, University of Missouri, Columbia, MO, USA; 4 Metabolic Technologies Inc, Iowa State University Research Park, Ames, IA, USA; 5 AP Nutrition LLC, Missoula, MT, USA; 6 Increnovo LLC, Milwaukee, WI, USA

## Introduction

Extracellular adenosine triphosphate (ATP) is hypothesized to stimulate vasodilation by binding to endothelial ATP/UTP-selective P2Y2 receptors; a phenomenon which is posited to be accelerated during exercise. Nonetheless, no studies to our knowledge have delineated if supplemental ATP enhances the blood flow response to exercise. Herein, we used a rat model to examine how different dosages of acute oral ATP administration affected the femoral blood flow response prior to, during, and after an exercise bout. In addition, we performed a single dose chronic administration study in resistance trained athletes.

## Methods

Animal study: After anesthesia male Wistar rats (~ 300 g) were placed under isoflurane anesthesia and subsequently gavage-fed either 0.003 g (100 mg, species and body surface area-adjusted human equivalent dosage, n=4), 0.012 g (400 mg, n=4), 0.031 g (1,000 mg, n=5), or 0.049 g (1,600 mg, n=5) of crystallized oral ATP disodium salt (Peak ATP®, TSI, Missoula, MT); rats that were not gavage-fed were used as controls (n=5). A blood flow probe was placed on the proximal portion of the right femoral artery and stimulation electrodes were placed in the right gastrocnemius muscle for an electrically-evoked plantarflexion exercise bout. Blood flow was then monitored continuously: a) 60 min prior to an electrically-evoked leg-kicking exercise (180 contractions), b) during and c) 90 min following the leg-kicking exercise. Areas under the pre-exercise, exercise, post-exercise, and total blood flow curves (AUC) were compared among conditions using one-way ANOVAs.

Human Study: In a pilot study, 12 college-aged resistance-trained participants were randomly assigned to an ATP or no ATP group. During week one, subjects were given no ATP, and 400 mg of ATP daily for 12 weeks, and prior to an acute arm exercise bout (60 biceps curl contractions) at weeks 1, 4, 8, and 12. Ultrasonography determined volumetric blood flow and vessel dialation in the brachial artery was measured at rest before taking the supplement and 30 minutes after at rest, and then at 0, 3, and 6 minutes after the exercise.

## Results

Animal Study: Rats fed 0.031 g (1000 mg human equivalent dosage) demonstrated significantly greater recovery blood flow (p = 0.007) and total blood flow AUC values (p = 0.048) compared to CTL rats. Specifically, blood flow was elevated in rats fed 0.031 g versus CTL rats at 20 to 90 min post exercise when examining 10-min blood flow intervals (p < 0.05). When examining within-group differences relative to baseline values, rats fed the 0.031 g (1,000 mg) and 0.049 g (1,600 mg) dosages exhibited the most robust increases in blood flow during exercise and into the recovery period.

Human study: At weeks 1, 8, and 12 there were significant differences in blood flow at 0, and 3 minutes post exercise in the ATP supplemented relative to the control week (wk 0-No ATP), along with significant elevations in brachial dilation at those time points.

## Conclusions

These are the first data to our knowledge to demonstrate that oral ATP administration can increase blood flow, and is particularly effective during exercise recovery.

